# Pathomx: an interactive workflow-based tool for the analysis of metabolomic data

**DOI:** 10.1186/s12859-014-0396-9

**Published:** 2014-12-10

**Authors:** Martin A Fitzpatrick, Catherine M McGrath, Stephen P Young

**Affiliations:** Rheumatology Research Group, Centre for Translational Inflammation Research, College of Medical and Dental Sciences, University of Birmingham, Birmingham, B15 2WD UK

**Keywords:** *Metabolomics*, *Omics*, *nmr*, *Analysis*, *Visualisation*, *Workflow*, *Automation*, *Python*

## Abstract

**Background:**

Metabolomics is a systems approach to the analysis of cellular processes through small-molecule metabolite profiling. Standardisation of sample handling and acquisition approaches has contributed to reproducibility. However, the development of robust methods for the analysis of metabolomic data is a work-in-progress. The tools that do exist are often not well integrated, requiring manual data handling and custom scripting on a case-by-case basis. Furthermore, existing tools often require experience with programming environments such as MATLAB® or R to use, limiting accessibility. Here we present Pathomx, a workflow-based tool for the processing, analysis and visualisation of metabolomic and associated data in an intuitive and extensible environment.

**Results:**

The core application provides a workflow editor, IPython kernel and a HumanCyc™-derived database of metabolites, proteins and genes. Toolkits provide reusable tools that may be linked together to create complex workflows. Pathomx is released with a base set of plugins for the import, processing and visualisation of data. The IPython backend provides integration with existing platforms including MATLAB® and R, allowing data to be seamlessly transferred. Pathomx is supplied with a series of demonstration workflows and datasets. To demonstrate the use of the software we here present an analysis of 1D and 2D ^1^H NMR metabolomic data from a model system of mammalian cell growth under hypoxic conditions.

**Conclusions:**

Pathomx is a useful addition to the analysis toolbox. The intuitive interface lowers the barrier to entry for non-experts, while scriptable tools and integration with existing tools supports complex analysis. We welcome contributions from the community.

**Electronic supplementary material:**

The online version of this article (doi:10.1186/s12859-014-0396-9) contains supplementary material, which is available to authorized users.

## Background

Metabolomics is a systems approach to the analysis of cellular processes through small-molecule metabolite profiles of a cell, tissue, organ or organism that results from the combined action of proteome, transcriptome and genome [1]. Metabolomics can be split broadly into targeted and untargeted approaches. Targeted metabolomics uses focused study of known pathways, reactions or metabolites in *in vitro* cell models and has been used to gain insight into metabolic requirements and vulnerabilities of cancer cells [2]. Untargeted metabolomics is a *hypothesis-forming* approach in which datasets derived from biological fluids are queried using multivariate analysis techniques, with the goal of identifying biomarkers or metabolic changes that can inform future study. This approach has been successfully employed for the identification of novel disease markers [3].

The standardisation of sample handling and data acquisition has contributed to improved reproducibility in metabolomics [4]. Data analysis methods in contrast are less well defined. Existing tools commonly build on mathematical environments, such as MATLAB® or R and require a level of familiarity not usually available in those from non-mathematical backgrounds. The difficulties moving data between these environments and associated packages is a hindrance to an integrated workflow. In our own group we have used this type of hybrid platform, combining MATLAB®-based NMRLab and MetaboLab [5] for processing and PLS Toolbox (Eigenvector Research, Wenatchee WA USA) for multivariate analysis, with Chenomx (Edmonton, Alberta, Canada) and the Human Metabolome Database [6] for metabolite identification. It is our experience that the complexity of the analysis workflow acts as a significant barrier to the use of metabolomics by non-experts, hinders discovery and slows throughput.

These issues are not unique to metabolomic analysis and the preceding decade has seen work to address them within the bioinformatics field. Scientific workflow tools have emerged in recent years as a powerful and flexible approach to the analysis of large datasets [7]. Automation of workflows can contribute to the reproducibility of analysis and reduction in error, while simultaneously increasing throughput. The major workflow analysis platforms in current use are Taverna [8] and Galaxy [9], which have established themselves as key tools in the bioinformaticians' toolkit. Both share a common approach of stepwise workflow-construction paired with server-based batch processing, yet differ on the level of abstraction of their components. Taverna is a low-level workflow creator, offering construction of complex functions from discrete algorithmic steps and with a particular focus on remote service integration. Galaxy in contrast offers high-level components that perform common bioinformatics tasks wholesale, with a focus on local-service integration and the need for no programming experience. Both platforms have been developed with a focus on genomic and transcriptomics analysis and lack support for the analysis of metabolomic data. The batch-based processing paradigm also limits application to the steps of analysis that can be fully automated while the latter stages of metabolomic data analysis are typically more exploratory, with iterative application of multivariate techniques, interrogation of biological databases, and pathway visualisation for interpretation of the data. Tools are already available to aid in the various stages of metabolomic data analysis, with MetaboAnalyst [10], a web-based metabolomic analysis pipeline, being of particular note. It includes modules for enrichment, pathway and time-series analysis, and has a particular focus on usability with the complete pipeline configurable through a simple web-based interface. However, this simplicity does come at the cost of the adaptability and automation that workflow analysis can offer. Further, the inability to adapt or extend analysis modules means that complete analysis of a dataset will often require other tools.

Recognising the benefits that workflow-based analysis could offer to metabolomics analysis while hoping to overcome the limitations of batch-based processing, we developed Pathomx: a workflow-based tool for data analysis. The software is designed to be adaptable, intuitive and to integrate well with existing tools and pipelines, acting as the essential glue in the metabolomics toolbox.

## Implementation

Pathomx is an open source and cross-platform analysis tool. It is developed in Python (v2.7; Python Software Foundation) with a graphical user interface (GUI) based on Qt (v5.1; Digia) and graphing powered by Matplotlib (v1.1.1) [11]. The processing kernel is based on IPython (v3.0.0).

In Pathomx nomenclature *plugins* provide *tools* that are then used for construction of *workflows*. The software ships with a base set of tools for data import, processing, analysis, visualisation and export based on the NumPy (v1.7.1), SciPy (v0.12.0) [12], Pandas (v0.14.1), SkiKit-Learn (v0.15.1) [13] and NMRGlue (v.0.4) [14] Python packages. Many of the algorithms in the default toolkit have subsequently been released as standalone Python packages to allow use outside Pathomx. These include biocyc (v0.1.0) a Python BioCyc API, gpml2svg (v0.3.0) a GPML renderer, icoshift (v0.6.0) a Python implementation of the Icoshift algorithm [15], metaboviz (v0.0.3) a metabolic pathway drawing package utilising the pydot (v1.0.28) interface to Graphviz (v2.12) [16] and pathminer (v0.0.2) a metabolic pathway mining algorithm. The functionality described in this paper relates to the base plugins provided with Pathomx 3.0.

Data analysis workflows are constructed using a drag-and-drop interface. Dragging a tool from the toolkit creates a new tool in the workflow. Selecting the tool allows configuration options to be changed, data sources to be configured and the tool code to be run. Inputs can also be managed directly from the workflow editor by dragging the output of one tool into the appropriate input of another. Recalculation and regeneration of figures is dynamic and the current run-state is visualised within the editor (blue = complete, red = error, green = active). The default toolkit makes extensive use of Pandas DataFrames and standard structures to allow tools to communicate easily. Tools can make use of parallel processing to allow efficient execution of complex workflows on large datasets on a standard modern desktop machine. Errors are flagged with both descriptive text and kernel backtraces for debugging purposes. Source code is available for all tools and can be modified using the inline editor to tweak behaviour. Resulting workflows can be exported as standalone scripts to run independently of the Pathomx environment. Figures can be exported as high-resolution TIF files for publication. Interfaces to both MATLAB® and R are available through the IPython backend allowing data to be passed between environments as required. Data may be imported from a number of other tools and public databases, including Metabolights [17] and Gene Expression Omnibus (GEO) [18].

Pathomx includes a subset of the HumanCyc™ *Homo sapiens* pathway data available under license from SRI International [19]. Database cross-referencing is supported for KEGG [20], HMDB [6] and other databases, generated from BioCyc annotations and the MNXref database [21].

## Results and discussion

**Figure 1:**
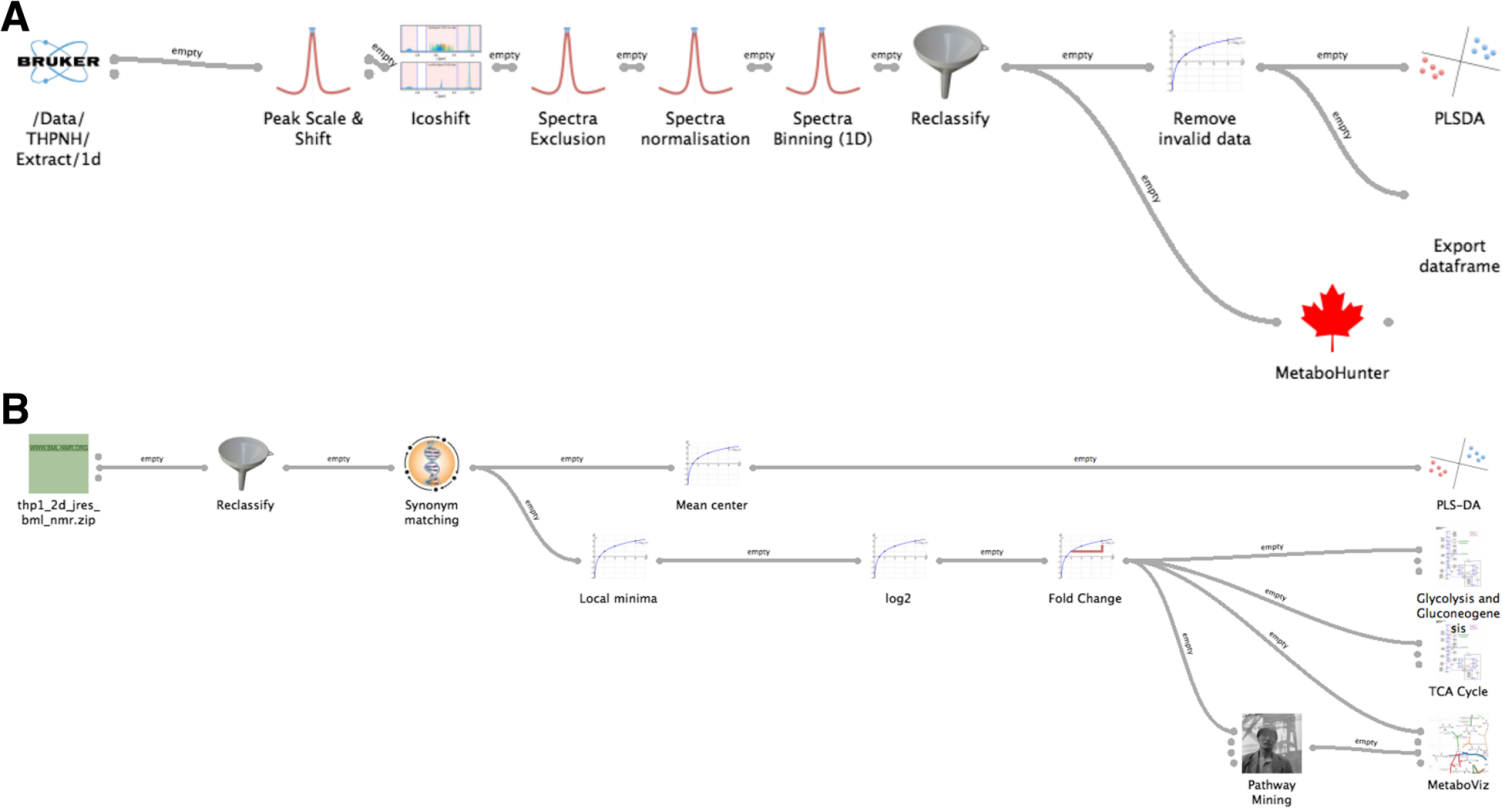
**Graphical representation of the Pathomx workflows used in the generation of the outputs.** **A.** 1D 1H Bruker NMR analysis workflow. **B.** 2D JRES BML-NMR analysis workflow. Both workflows are included in the additional resources and may be used to re-process the supplementary data. Workflows are constructed through a drag-and-drop interface.

To demonstrate the use of the Pathomx software we here present a sample workflow and metabolomic dataset. Sample data are derived from the culture of the THP-1 human macrophage cell line under 20% and 1% O_2_ conditions for 24 hours. Intracellular metabolites were isolated by standard methods [22] and 1D and 2D J-resolved (JRES) ^1^H NMR spectra subsequently acquired on a 600 MHz B600 Bruker Avance III spectrometer with TCI 1.7 mm z-PFG cryogenic probe. 2D JRES spectra were quantified and metabolites identified using the Field Independent Metabolite Analysis (FIMA) Birmingham Metabolite Library (BML) [23] at bml-nmr.org. The resulting data files were loaded into Pathomx and a workflow constructed using the default toolkit (Figure 1A and Figure 1B). Data files and workflows are provided with this paper (Additional file 1, Additional file 2, Additional file 3 and Additional file 4).

### Spectral processing

Prerequisite to the analysis of 1D NMR data are a number of spectral processing steps that ensure that any observed variation in the data is reflective of biology. An example 1D NMR analysis workflow is included that performs these steps with the included Bruker-format NMR output. 1D NOESY ^1^H NMR spectra are first peak-aligned using a TMSP reference peak and then spectra were further aligned using the Icoshift correlation-shifting segmental alignment algorithm. Spectra were then binned at 0.015 ppm and assigned to experimental groups (Figure 1).

### Metabolite identification

Identification and quantification of the 2D JRES data was performed using the BML-NMR service and the resulting data can be loaded automatically into Pathomx. Identification of metabolites in 1D data is typically more involved and Pathomx includes support for both manual peak assignment and automated peak-metabolite quantification with Chenomx. However, in the provided workflow we have used MetaboHunter [24] a free remote web service which identifies metabolites using peak-matching to the HMDB (Figure 4A).

### Multivariate analysis

Metabolomic datasets are commonly analysed using multivariate methods. Pathomx provides support for two common methods: principal components analysis (PCA) and partial least squares discriminant analysis (PLS-DA). PLS-DA is suited to the analysis of distinct groups as in this dataset—20% O_2_ (N), 1% O_2_ (H) — and is included in both the 1D and 2D workflows. The resulting loadings plot show the contributions of each peak, metabolite or region of spectra to the separation between the groups (Figure 4).

**Figure 2:**
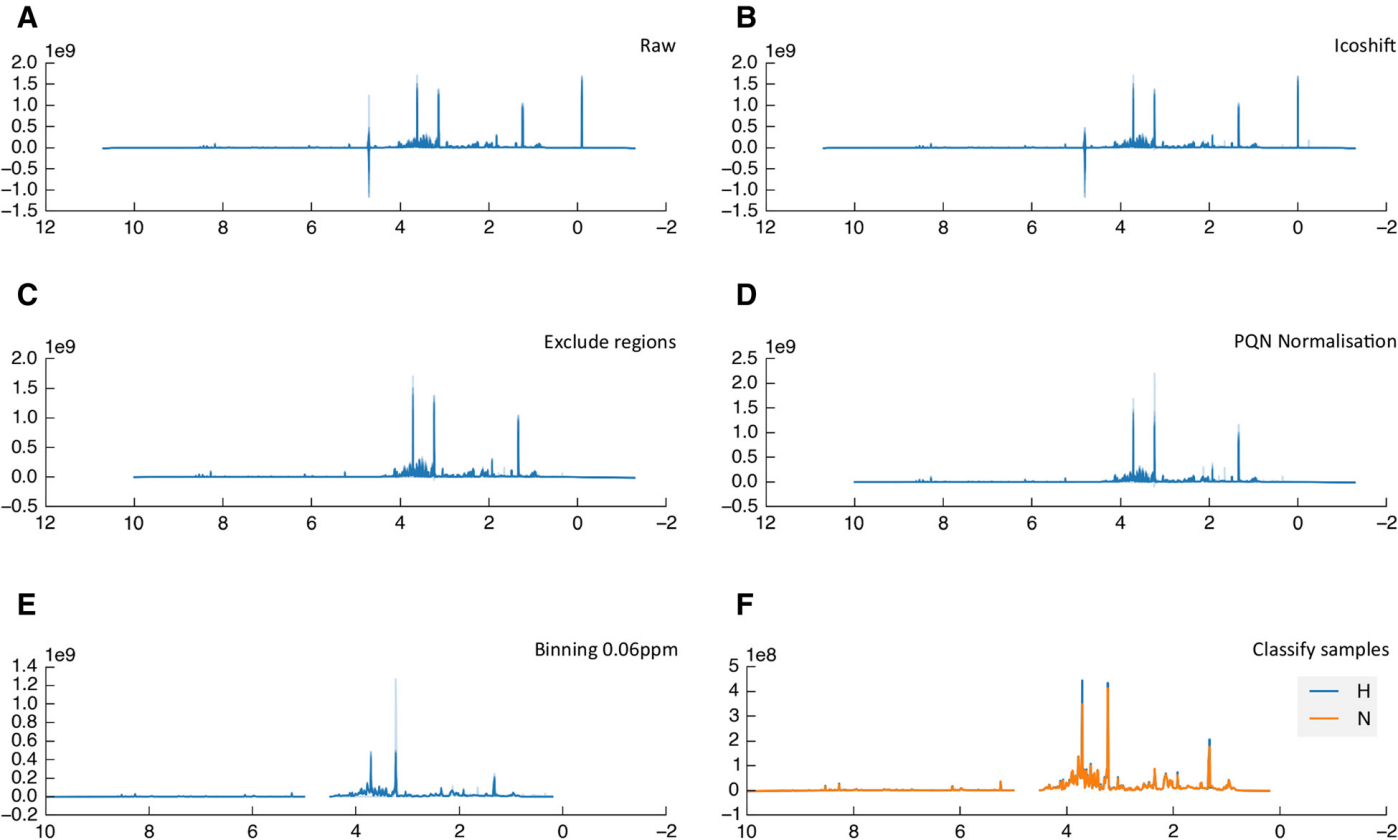
**Example outputs from the 1D processing workflow.** **A.** Raw ^1^H NMR spectra. **B.** Aligned using Icoshift. **C.** TMSP and water regions are excluded. **D.** PQN normalisation. **E.** Spectral binning to 0.06 ppm. **F.** Mapped to appropriate classification groups by spectra number - normoxia (orange) hypoxia (blue).

**Figure 3:**
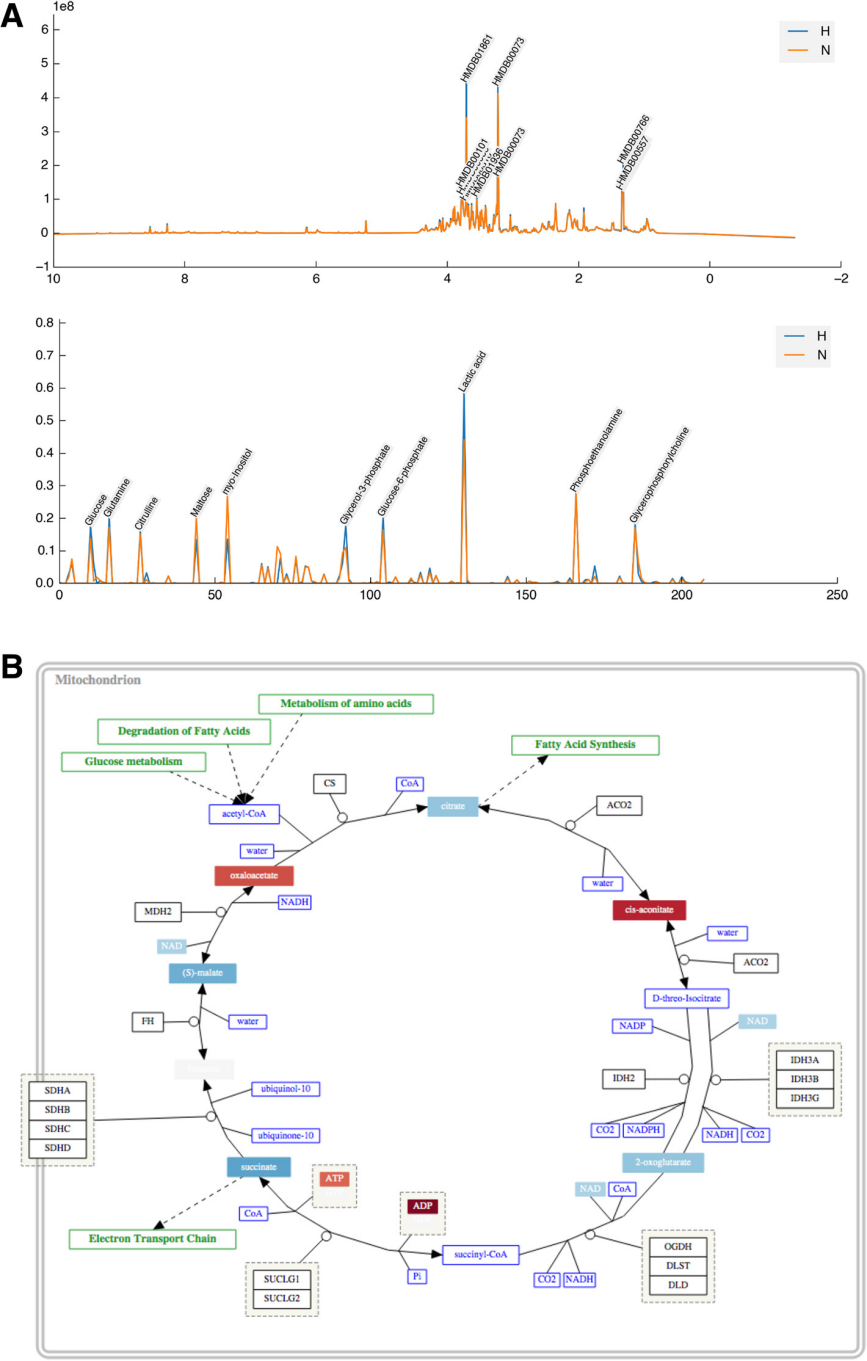
**Metabolite identification generated from MetaboHunter and BML-NMR.** **A.** 1D NMR spectra with peaks matched to HMDB identifiers by MetaboHunter. **B.** 2D JRES BML-NMR quantified metabolites. The default toolkit also supports manual peak assignment and import of quantification data from Chenomx. Identified metabolites can be annotated onto all subsequent plots and used for pathway analysis.

### Metabolic pathway analysis

Metabolic pathway analysis allows changes in metabolites to be mapped onto pathways for visualisation and interpretation. To generate pathway maps, fold change values for identified metabolites were calculated between 20% O_2_ and 1% O_2_ cultures. These can be visualised directly on standard GPML/Wikipathways pathway maps (Figure 3B). In experiments where the pathway changes cannot be predicted, the included pathway mining tools can be used to select and visualise the most up- and down- regulated pathways from the data. This uses the included BioCyc API and cached data to map quantified metabolites to relevant pathways. The resulting top 5 pathways are visualised in the MetaboViz automated pathway-drawing tool, with fold-change data for each metabolite represented on a blue-red colour scale (Figure 5). Under normal oxygenated conditions, glucose is metabolised through the glycolytic pathway ending in the production of pyruvate, which can in turn feed into the TCA cycle. The TCA cycle, while not oxygen dependent itself, requires recycling of NADH to NAD by the oxygen-dependent electron-transport chain to maintain function. In the absence of oxygen, NADH is not recycled and the TCA cycle is impeded. The Pathomx pathway-mining analysis of our model-system correctly identifies this regulation, ranking the TCA cycle as most-altered pathway in the system. Pathway-based visualisation shows down-regulation of a majority of TCA cycle metabolites, together with low NAD concentration as an indicator of oxygen-dependent electron-transport chain failure. In the absence of the feed forward into the TCA cycle, excess pyruvate is excreted as lactate.

**Figure 4:**
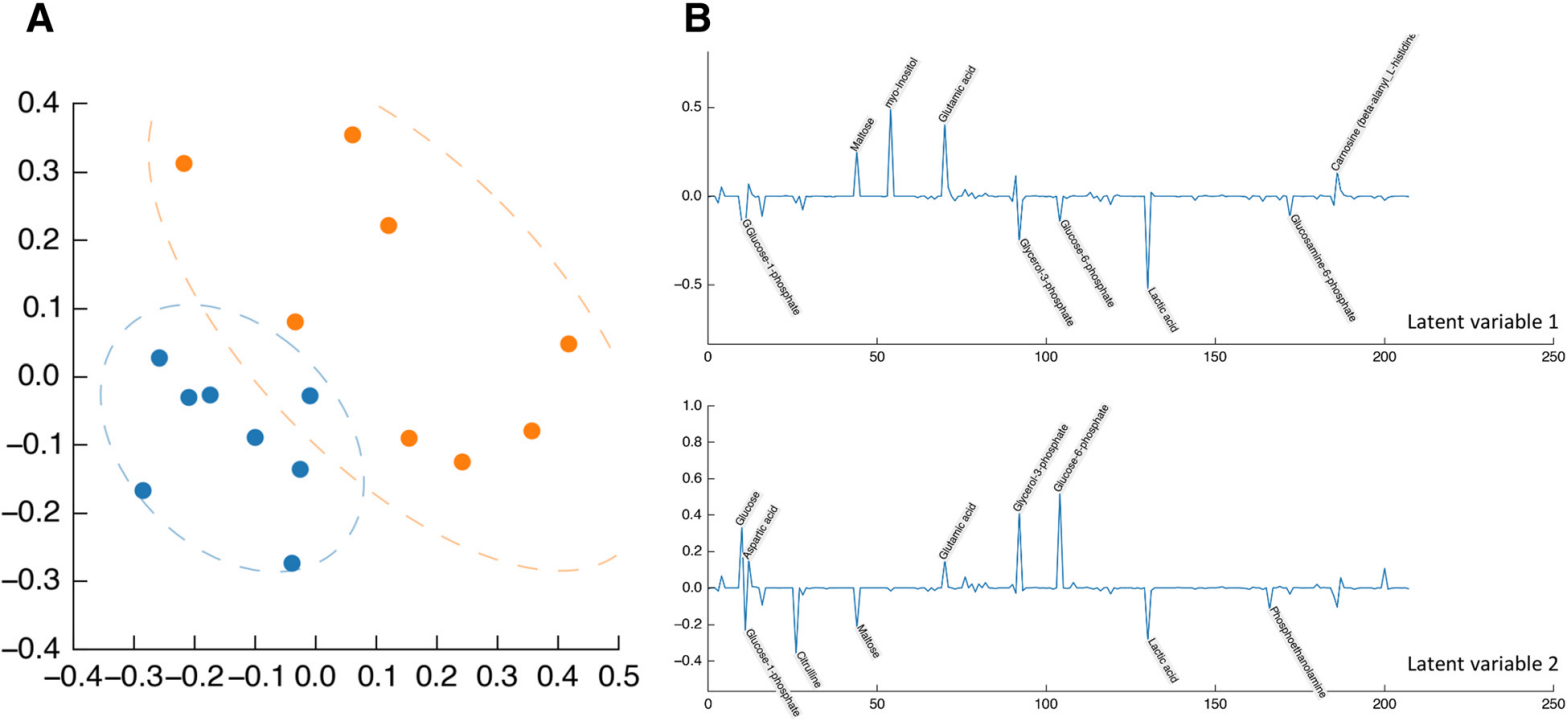
**The default toolkit includes support for multivariate analysis. A.** A PLS-DA showing separation between the two groups in the 2D JRES PQN dataset, showing normoxia (orange) hypoxia (blue) class groups. The first two latent variables are shown alongside, with metabolite annotations visible. THP-1 cells under hypoxia produce more lactate. **B.** GPML/WikiPathways pathway of the TCA cycle showing fold-change differences between normoxia and hypoxia visualised on a red-blue scale showing up and down-regulated metabolites respectively.

**Figure 5:**
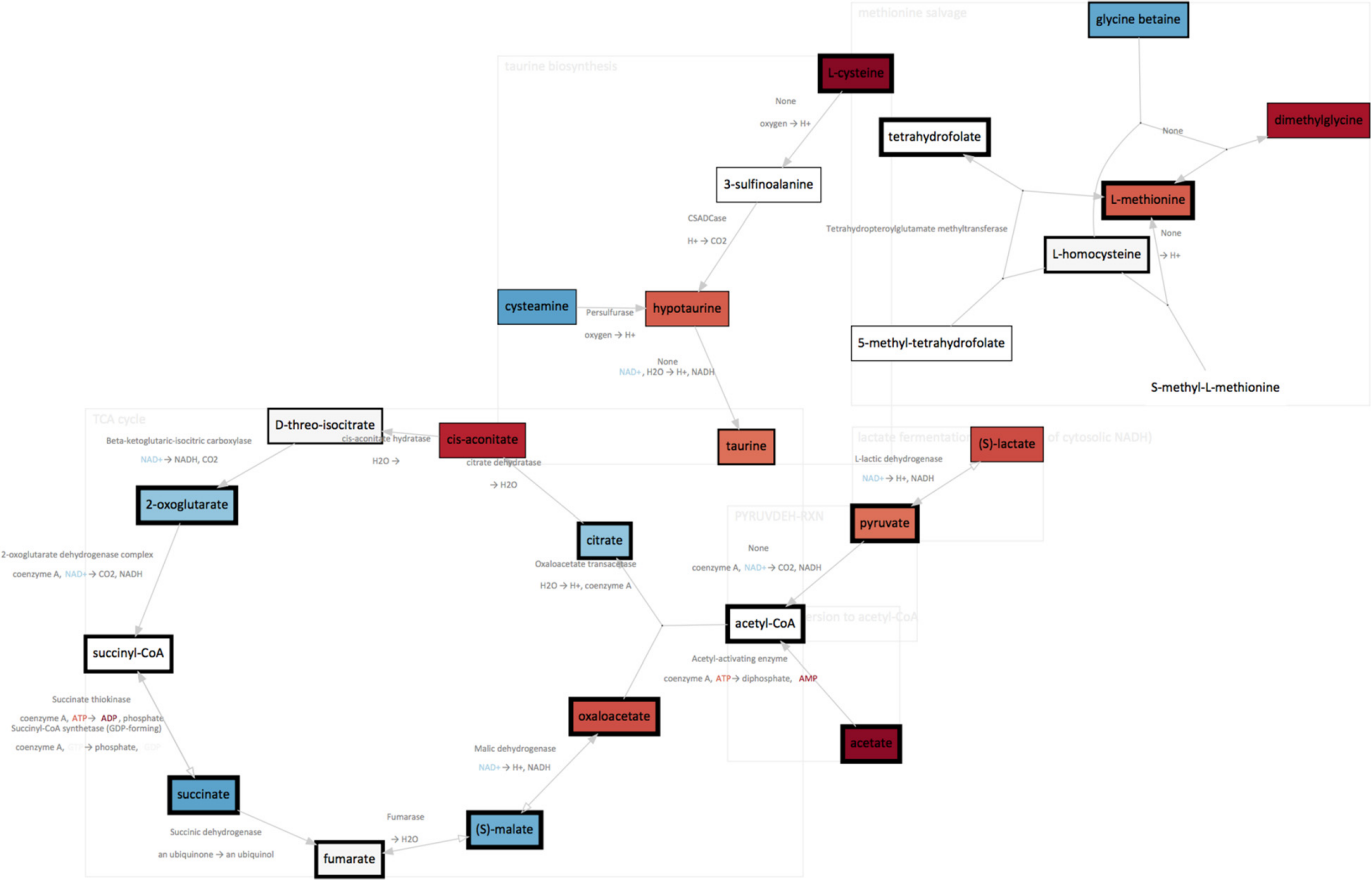
**Pathway mined metabolites as visualised by MetaboViz plugin.** Pathways identified by the Pathway Mining plugin and rendered using the included MetaViz plugin. Changes are visualised on a red-blue scale, showing up- and down-regulated metabolites respectively. Analysis shows down-regulation of TCA cycle metabolites, together with low NAD as an indicator of oxygen-dependent electron-transport chain failure.

## Conclusions

Pathomx is a workflow-based tool for the exploration of metabolomic data. It supports a complete processing workflow through data import, processing, analysis and visualisation. It is open source and features a plugin system that can be readily extended with new features and integrates readily with existing tools. Workflow construction requires no prior programming knowledge but can utilise it where available. The resulting workflows can be shared and re-used or exported as standalone Python scripts. Plugin development is supported through a simple, well-documented Python-based API. We welcome contributions of plugins and workflows from the community.

## Availability and requirements

**Project name:** Pathomx

**Project home page:**http://pathomx.org

**Platform:** Binaries are available for download on Windows and MacOS X. Installation on Linux (Ubuntu) is supported via PyPi. Source code is available.

Programming language: Python 2.7, Qt 5.2

**Other requirements:** Package binary contains all requirements

**License:** GNU GPL v3.0

**Any restrictions to use by non-academics:** N/A

## Additional files

Additional file 1:
**1D THP-1 Pathomx analysis workflow.** Pathomx analysis workflow for 1D ^1^H Bruker NMR data, acquired from the THP-1 normoxia (N) and hypoxia (H) experiment. This file contains the settings for each processing step and workflow connections between them. Loading the relevant data into this workflow will result in the same outputs presented in the paper, which can be explored in further detail. Additional file 2:
**1D Raw Bruker NMR dataset – THP-1 N&H.** Experimental data from THP-1 normoxia (N) and hypoxia (H) experiment acquired by 1D ^1^H NOESY NMR on Bruker spectrometer. Additional file 3:
**2D THP-1 Pathomx analysis workflow.** Pathomx analysis workflow for 2D BML-NMR processed data, acquired from the THP-1 normoxia (N) and hypoxia (H) experiment . This file contains the settings for each processing step and workflow connections between them. Loading the relevant data into this workflow will result in the same outputs presented in the paper, which can be explored in further detail. Additional file 4:
**2D Processed NMR dataset – THP-1 N&H.** Experimental data from THP-1 normoxia (N) and hypoxia (H) experiment acquired by 2D ^1^H JRES NMR on Bruker spectrometer and processed via the BML-NMR web service.

## References

[1] Goodacre R, Vaidyanathan S, Dunn WB, Harrigan GG, Kell DB (2004). Metabolomics by numbers: acquiring and understanding global metabolite data. Trends Biotechnol.

[2] Armitage EG, Barbas C: **Metabolomics in cancer biomarker discovery: Current trends and future perspectives.** *J Pharm. Biomed Anal* 2013. 10.1016/j.jpba.2013.08.041 24091079

[3] Fitzpatrick M, Young SP (2013). Metabolomics - a novel window into inflammatory disease. Swiss Med Wkly.

[4] Salek RM, Haug K, Conesa P, Hastings J, Williams M, Mahendraker T, Maguire E, González-Beltrán AN, Rocca-Serra P, Sansone S-A, Steinbeck C (2013). The MetaboLights repository: curation challenges in metabolomics. Database (Oxford).

[5] Ludwig C, Günther UL (2011). MetaboLab–advanced NMR data processing and analysis for metabolomics. BMC Bioinformatics.

[6] Wishart DS, Jewison T, Guo AC, Wilson M, Knox C, Liu Y, Djoumbou Y, Mandal R, Aziat F, Dong E, Bouatra S, Sinelnikov I, Arndt D, Xia J, Liu P, Yallou F, Bjorndahl T, Perez-Pineiro R, Eisner R, Allen F, Neveu V, Greiner R, Scalbert A (2013). HMDB 3.0–The Human Metabolome Database in 2013. Nucleic Acids Res.

[7] Curcin V, Ghanem M (2008). Scientific workflow systems - can one size fit all?. Cairo Int Biomed Eng Conf.

[8] Oinn T, Addis M, Ferris J, Marvin D, Senger M, Greenwood M, Carver T, Glover K, Pocock MR, Wipat A, Li P (2004). Taverna: a tool for the composition and enactment of bioinformatics workflows. Bioinformatics.

[9] Giardine B, Riemer C, Hardison RC, Burhans R, Elnitski L, Shah P, Zhang Y, Blankenberg D, Albert I, Taylor J, Miller W, Kent WJ, Nekrutenko A (2005). Galaxy: a platform for interactive large-scale genome analysis. Genome Res.

[10] Xia J, Mandal R, Sinelnikov IV, Broadhurst D, Wishart DS (2012). MetaboAnalyst 2.0-a comprehensive server for metabolomic data analysis. Nucleic Acids Res.

[11] Hunter JD (2007). Matplotlib: a 2D graphics environment. Comput Sci Eng.

[12] Oliphant TE (2007). Python for scientific computing. Comput Sci Eng.

[13] Pedregosa F, Weiss R, Brucher M (2011). Scikit-learn: machine learning in python. J Mach Learn Res.

[14] Helmus JJ, Jaroniec CP (2013). Nmrglue: an open source Python package for the analysis of multidimensional NMR data. J Biomol NMR.

[15] Savorani F, Tomasi G, Engelsen SB (2010). Icoshift: a versatile tool for the rapid alignment of 1D NMR spectra. J Magn Reson.

[16] Gansner E, North SC (1999). An open graph visualization system and its applications to software engineering. Softw - Pract Exp.

[17] Haug K, Salek RM, Conesa P, Hastings J, de Matos P, Rijnbeek M, Mahendraker T, Williams M, Neumann S, Rocca-Serra P, Maguire E, González-Beltrán A, Sansone S-A, Griffin JL, Steinbeck C (2013). MetaboLights–an open-access general-purpose repository for metabolomics studies and associated meta-data. Nucleic Acids Res.

[18] Edgar R, Domrachev M, Lash AE (2002). Gene Expression Omnibus: NCBI gene expression and hybridization array data repository. Nucleic Acids Res.

[19] Trupp M, Altman T, Fulcher CA, Caspi R, Krummenacker M, Paley S, Karp PD (2010). Beyond the genome ( BTG ) is a ( PGDB ) pathway genome databasets. HumanCyc.

[20] Kanehisa M, Goto S, Sato Y, Furumichi M, Tanabe M (2012). KEGG for integration and interpretation of large-scale molecular data sets. Nucleic Acids Res.

[21] Bernard T, Bridge A, Morgat A, Moretti S, Xenarios I, Pagni M (2014). Reconciliation of metabolites and biochemical reactions for metabolic networks. Brief Bioinform.

[22] Wu H, Southam AD, Hines A, Viant MR (2008). High-throughput tissue extraction protocol for NMR- and MS-based metabolomics. Anal Biochem.

[23] Ludwig C, Easton J, Lodi A, Tiziani S, Manzoor S, Southam A, Byrne J, Bishop L, He S, Arvanitis T, Günther U, Viant M (2012). Birmingham Metabolite Library: a publicly accessible database of 1-D 1H and 2-D 1H J-resolved NMR spectra of authentic metabolite standards (BML-NMR). Metabolomics.

[24] Tulpan D, Léger S, Belliveau L, Culf A, Čuperlović-Culf M (2011). MetaboHunter: an automatic approach for identification of metabolites from 1H-NMR spectra of complex mixtures. BMC Bioinformatics.

