# Supplementary figures and images for: Pathomx: an interactive workflow-based tool for the analysis of metabolomic data
Source: BMC Bioinformatics. 2014 Dec 10;15(1):396. doi: 10.1186/s12859-014-0396-9 (PMC4271363; doi:10.1186/s12859-014-0396-9)

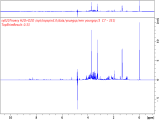

Supplement: Additional file 2: — 1D Raw Bruker NMR dataset – THP-1 N&H. Experimental data from THP-1 normoxia (N) and hypoxia (H) experiment acquired by 1D 1H NOESY NMR on Bruker spectrometer. [file 12859_2014_396_MOESM2_ESM.zip › 101/pdata/1/thumb.png]

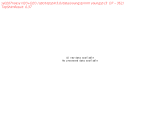

Supplement: Additional file 2: — 1D Raw Bruker NMR dataset – THP-1 N&H. Experimental data from THP-1 normoxia (N) and hypoxia (H) experiment acquired by 1D 1H NOESY NMR on Bruker spectrometer. [file 12859_2014_396_MOESM2_ESM.zip › 103/pdata/1/thumb.png]

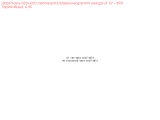

Supplement: Additional file 2: — 1D Raw Bruker NMR dataset – THP-1 N&H. Experimental data from THP-1 normoxia (N) and hypoxia (H) experiment acquired by 1D 1H NOESY NMR on Bruker spectrometer. [file 12859_2014_396_MOESM2_ESM.zip › 105/pdata/1/thumb.png]

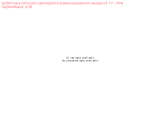

Supplement: Additional file 2: — 1D Raw Bruker NMR dataset – THP-1 N&H. Experimental data from THP-1 normoxia (N) and hypoxia (H) experiment acquired by 1D 1H NOESY NMR on Bruker spectrometer. [file 12859_2014_396_MOESM2_ESM.zip › 107/pdata/1/thumb.png]

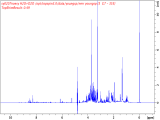

Supplement: Additional file 2: — 1D Raw Bruker NMR dataset – THP-1 N&H. Experimental data from THP-1 normoxia (N) and hypoxia (H) experiment acquired by 1D 1H NOESY NMR on Bruker spectrometer. [file 12859_2014_396_MOESM2_ESM.zip › 109/pdata/1/thumb.png]

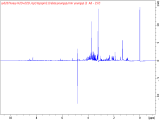

Supplement: Additional file 2: — 1D Raw Bruker NMR dataset – THP-1 N&H. Experimental data from THP-1 normoxia (N) and hypoxia (H) experiment acquired by 1D 1H NOESY NMR on Bruker spectrometer. [file 12859_2014_396_MOESM2_ESM.zip › 113/pdata/1/thumb.png]

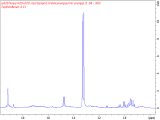

Supplement: Additional file 2: — 1D Raw Bruker NMR dataset – THP-1 N&H. Experimental data from THP-1 normoxia (N) and hypoxia (H) experiment acquired by 1D 1H NOESY NMR on Bruker spectrometer. [file 12859_2014_396_MOESM2_ESM.zip › 119/pdata/1/thumb.png]

expno 100. Title=

. Experimental (blue), fitted (yellow) and difference (fitted-exp, red) sum pJRES spectra

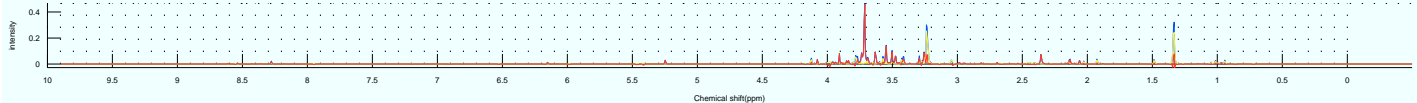

Supplement: Additional file 4: — 2D Processed NMR dataset – THP-1 N&H. Experimental data from THP-1 normoxia (N) and hypoxia (H) experiment acquired by 2D 1H JRES NMR on Bruker spectrometer and processed via the BML-NMR web service. [file 12859_2014_396_MOESM4_ESM.zip › BMLJOB_338/indiv_expno_outputs/100/graph_out_expno_100.pdf]

expno 102. Title= . Experimental (blue), fitted (yellow) and difference (fitted-exp, red) sum pJRES spectra

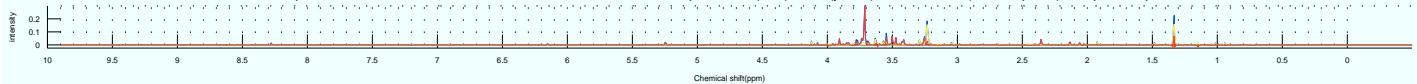

Supplement: Additional file 4: — 2D Processed NMR dataset – THP-1 N&H. Experimental data from THP-1 normoxia (N) and hypoxia (H) experiment acquired by 2D 1H JRES NMR on Bruker spectrometer and processed via the BML-NMR web service. [file 12859_2014_396_MOESM4_ESM.zip › BMLJOB_338/indiv_expno_outputs/102/graph_out_expno_102.pdf]

expno 104. Title= . Experimental (blue), fitted (yellow) and difference (fitted-exp, red) sum pJRES spectra

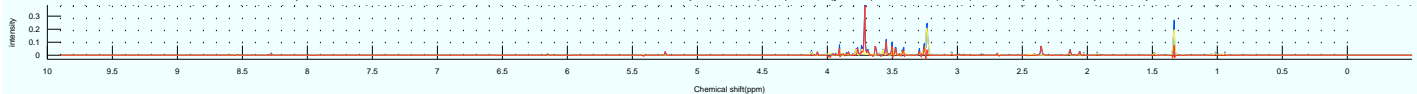

Supplement: Additional file 4: — 2D Processed NMR dataset – THP-1 N&H. Experimental data from THP-1 normoxia (N) and hypoxia (H) experiment acquired by 2D 1H JRES NMR on Bruker spectrometer and processed via the BML-NMR web service. [file 12859_2014_396_MOESM4_ESM.zip › BMLJOB_338/indiv_expno_outputs/104/graph_out_expno_104.pdf]

expno 106. Title= . Experimental (blue), fitted (yellow) and difference (fitted-exp, red) sum pJRES spectra

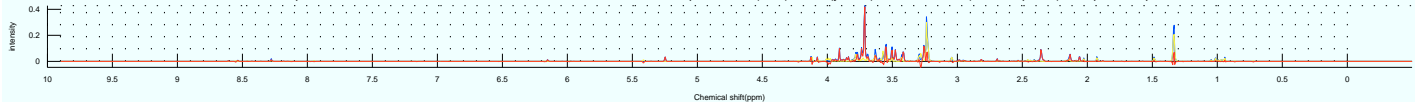

Supplement: Additional file 4: — 2D Processed NMR dataset – THP-1 N&H. Experimental data from THP-1 normoxia (N) and hypoxia (H) experiment acquired by 2D 1H JRES NMR on Bruker spectrometer and processed via the BML-NMR web service. [file 12859_2014_396_MOESM4_ESM.zip › BMLJOB_338/indiv_expno_outputs/106/graph_out_expno_106.pdf]

expno 108. Title=

. Experimental (blue), fitted (yellow) and difference (fitted-exp, red) sum pJRES spectra

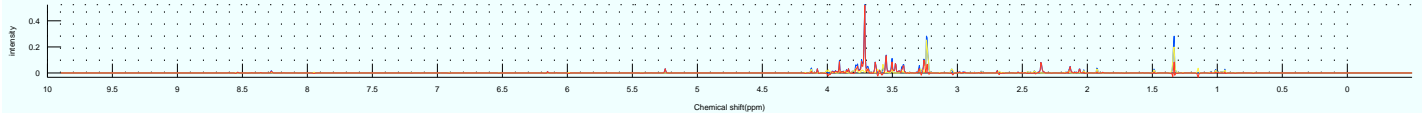

Supplement: Additional file 4: — 2D Processed NMR dataset – THP-1 N&H. Experimental data from THP-1 normoxia (N) and hypoxia (H) experiment acquired by 2D 1H JRES NMR on Bruker spectrometer and processed via the BML-NMR web service. [file 12859_2014_396_MOESM4_ESM.zip › BMLJOB_338/indiv_expno_outputs/108/graph_out_expno_108.pdf]

expno 110. Title= . Experimental (blue), fitted (yellow) and difference (fitted-exp, red) sum pJRES spectra

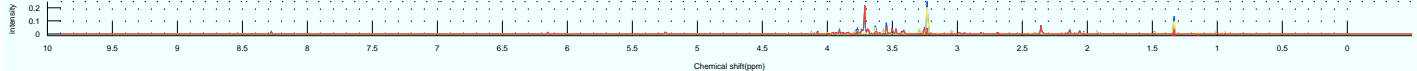

Supplement: Additional file 4: — 2D Processed NMR dataset – THP-1 N&H. Experimental data from THP-1 normoxia (N) and hypoxia (H) experiment acquired by 2D 1H JRES NMR on Bruker spectrometer and processed via the BML-NMR web service. [file 12859_2014_396_MOESM4_ESM.zip › BMLJOB_338/indiv_expno_outputs/110/graph_out_expno_110.pdf]

expno 112. Title= . Experimental (blue), fitted (yellow) and difference (fitted-exp, red) sum pJRES spectra

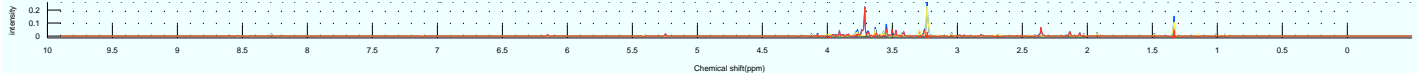

Supplement: Additional file 4: — 2D Processed NMR dataset – THP-1 N&H. Experimental data from THP-1 normoxia (N) and hypoxia (H) experiment acquired by 2D 1H JRES NMR on Bruker spectrometer and processed via the BML-NMR web service. [file 12859_2014_396_MOESM4_ESM.zip › BMLJOB_338/indiv_expno_outputs/112/graph_out_expno_112.pdf]

expno 116. Title= . Experimental (blue), fitted (yellow) and difference (fitted-exp, red) sum pJRES spectra

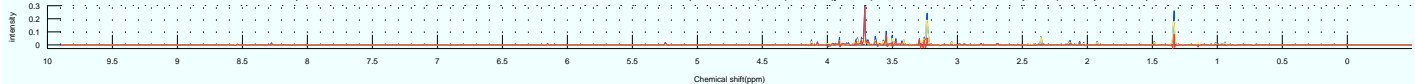

Supplement: Additional file 4: — 2D Processed NMR dataset – THP-1 N&H. Experimental data from THP-1 normoxia (N) and hypoxia (H) experiment acquired by 2D 1H JRES NMR on Bruker spectrometer and processed via the BML-NMR web service. [file 12859_2014_396_MOESM4_ESM.zip › BMLJOB_338/indiv_expno_outputs/116/graph_out_expno_116.pdf]

expno 118. Title= . Experimental (blue), fitted (yellow) and difference (fitted-exp, red) sum pJRES spectra

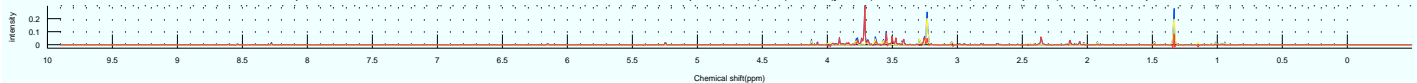

Supplement: Additional file 4: — 2D Processed NMR dataset – THP-1 N&H. Experimental data from THP-1 normoxia (N) and hypoxia (H) experiment acquired by 2D 1H JRES NMR on Bruker spectrometer and processed via the BML-NMR web service. [file 12859_2014_396_MOESM4_ESM.zip › BMLJOB_338/indiv_expno_outputs/118/graph_out_expno_118.pdf]

expno 120. Title= . Experimental (blue), fitted (yellow) and difference (fitted-exp, red) sum pJRES spectra

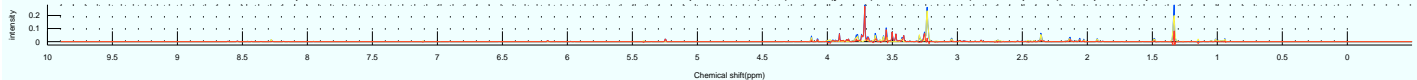

Supplement: Additional file 4: — 2D Processed NMR dataset – THP-1 N&H. Experimental data from THP-1 normoxia (N) and hypoxia (H) experiment acquired by 2D 1H JRES NMR on Bruker spectrometer and processed via the BML-NMR web service. [file 12859_2014_396_MOESM4_ESM.zip › BMLJOB_338/indiv_expno_outputs/120/graph_out_expno_120.pdf]

expno 86. Title= . Experimental (blue), fitted (yellow) and difference (fitted-exp, red) sum pJRES spectra

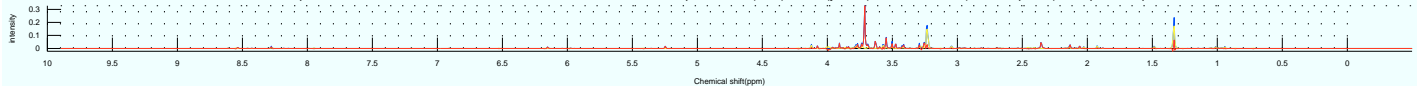

Supplement: Additional file 4: — 2D Processed NMR dataset – THP-1 N&H. Experimental data from THP-1 normoxia (N) and hypoxia (H) experiment acquired by 2D 1H JRES NMR on Bruker spectrometer and processed via the BML-NMR web service. [file 12859_2014_396_MOESM4_ESM.zip › BMLJOB_338/indiv_expno_outputs/86/graph_out_expno_86.pdf]

expno 90. Title= . Experimental (blue), fitted (yellow) and difference (fitted-exp, red) sum pJRES spectra

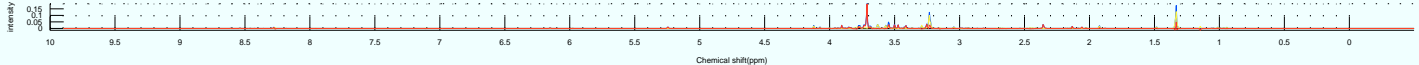

Supplement: Additional file 4: — 2D Processed NMR dataset – THP-1 N&H. Experimental data from THP-1 normoxia (N) and hypoxia (H) experiment acquired by 2D 1H JRES NMR on Bruker spectrometer and processed via the BML-NMR web service. [file 12859_2014_396_MOESM4_ESM.zip › BMLJOB_338/indiv_expno_outputs/90/graph_out_expno_90.pdf]

expno 92. Title= . Experimental (blue), fitted (yellow) and difference (fitted-exp, red) sum pJRES spectra

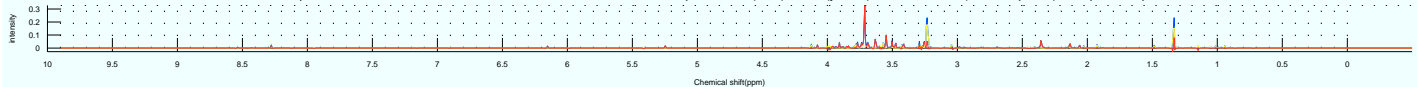

Supplement: Additional file 4: — 2D Processed NMR dataset – THP-1 N&H. Experimental data from THP-1 normoxia (N) and hypoxia (H) experiment acquired by 2D 1H JRES NMR on Bruker spectrometer and processed via the BML-NMR web service. [file 12859_2014_396_MOESM4_ESM.zip › BMLJOB_338/indiv_expno_outputs/92/graph_out_expno_92.pdf]

expno 94. Title= . Experimental (blue), fitted (yellow) and difference (fitted-exp, red) sum pJRES spectra

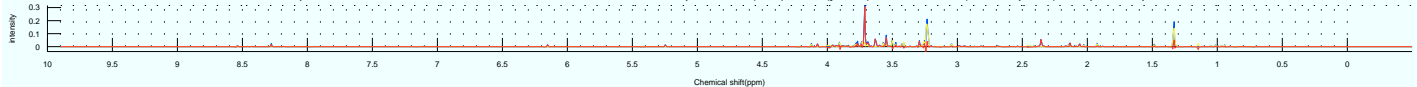

Supplement: Additional file 4: — 2D Processed NMR dataset – THP-1 N&H. Experimental data from THP-1 normoxia (N) and hypoxia (H) experiment acquired by 2D 1H JRES NMR on Bruker spectrometer and processed via the BML-NMR web service. [file 12859_2014_396_MOESM4_ESM.zip › BMLJOB_338/indiv_expno_outputs/94/graph_out_expno_94.pdf]

expno 96. Title= . Experimental (blue), fitted (yellow) and difference (fitted-exp, red) sum pJRES spectra

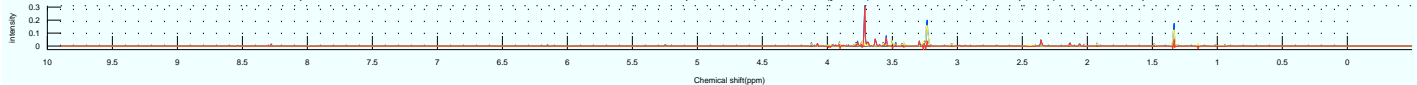

Supplement: Additional file 4: — 2D Processed NMR dataset – THP-1 N&H. Experimental data from THP-1 normoxia (N) and hypoxia (H) experiment acquired by 2D 1H JRES NMR on Bruker spectrometer and processed via the BML-NMR web service. [file 12859_2014_396_MOESM4_ESM.zip › BMLJOB_338/indiv_expno_outputs/96/graph_out_expno_96.pdf]

expno 98. Title=

. Experimental (blue), fitted (yellow) and difference (fitted-exp, red) sum pJRES spectra

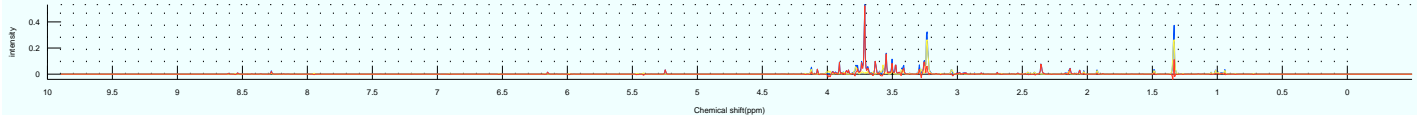

Supplement: Additional file 4: — 2D Processed NMR dataset – THP-1 N&H. Experimental data from THP-1 normoxia (N) and hypoxia (H) experiment acquired by 2D 1H JRES NMR on Bruker spectrometer and processed via the BML-NMR web service. [file 12859_2014_396_MOESM4_ESM.zip › BMLJOB_338/indiv_expno_outputs/98/graph_out_expno_98.pdf]
